# The Impact of Telemedicine on Rheumatology Care

**DOI:** 10.3389/fmed.2022.876835

**Published:** 2022-05-20

**Authors:** Wei Tang, Sean Inzerillo, Julia Weiner, Leila Khalili, Julia Barasch, Yevgeniya Gartshteyn, Maria Dall'Era, Cynthia Aranow, Meggan Mackay, Anca Askanase

**Affiliations:** ^1^Division of Rheumatology, Columbia University Irving Medical Center, New York, NY, United States; ^2^Columbia University Vagelos College of Physicians and Surgeons, New York, NY, United States; ^3^Lupus Clinic and Rheumatology Clinical Research Center, University of California, San Francisco, San Francisco, CA, United States; ^4^Feinstein Institutes for Medical Research, Manhasset, NY, United States

**Keywords:** autoimmune diseases (AD), telemedicine, quality of life, quality of care/care delivery, survey, telerheumatology

## Abstract

**Background:**

The pandemic disrupted the care of patients with rheumatic diseases; difficulties in access to care and its psychological impact affected quality of life. Telemedicine as an alternative to traditional face-to-face office visits has the potential to mitigate this impact.

**Objective:**

To evaluate patient and provider experience with telemedicine and its effect on care.

**Methods:**

We surveyed patients with rheumatic diseases and their rheumatology providers. The surveys were conducted in 2020 and repeated in 2021. We assessed data on quality of care and health-related quality of life.

**Results:**

Hundred patients and 17 providers responded to the survey. Patients reported higher satisfaction with telemedicine in 2021 compared to 2020 (94 vs. 84%), felt more comfortable with (96 vs. 86%), expressed a stronger preference for (22 vs. 16%), and higher intention to use telemedicine in the future (83 vs. 77%); patients thought physicians were able to address their concerns. While providers' satisfaction with telemedicine increased (18–76%), 14/17 providers believed that telemedicine visits were worse than in-person visits. There were no differences in annualized office visits and admissions. Mean EQ-5D score was 0.74, lower than general population (0.87) but equivalent to a subset of patients with SLE (0.74).

**Conclusion:**

Our data showed a high level of satisfaction with telemedicine. The lower rheumatology provider satisfaction raises concern if telemedicine constitutes an acceptable alternative to in-person care. The stable number of office visits, admissions, and the similar quality of life to pre-pandemic level suggest effective management of rheumatic diseases using telemedicine/in-person hybrid care.

## Introduction

Telemedicine, defined as the exchange of medical information through electronic communication to improve a patient's health, has long been utilized in rheumatology practice to maximize access to specialty care among populations in underserved areas and optimize healthcare delivery in routine clinical practice (1). Telemedicine encompasses a variety of formats using different technologies, including video conferences, telephone consultations, web-based conversations, and electronic messages (2).

During the height of the COVID-19 pandemic, rheumatic disease patients experienced significant health care interruption. Guaracha-Basáñez et al. reported that 51.3% patients with rheumatic diseases experienced health care interruption from March to June 2020 during the transition from in-person office visits to telemedicine (3). The increasing demand during the pandemic for healthcare resources, a soaring number of patients and limited physician availability galvanized the utilization of telemedicine in Rheumatology and other specialties (4). While in-person rheumatology practice was largely replaced by telemedicine to ensure the safety of both patients and providers, the implementation of telemedicine was challenging (5, 6).

It remains unclear whether telemedicine, especially the most commonly used video conference-based modality, can serve as a feasible alternative to conventional in-person clinical visits while achieving patient/provider satisfaction and maintaining the quality of care. Data on the quality of Rheumatology care delivered *via* telemedicine is sparse. De Thurah et al. randomized 294 patients to patient-reported outcome rheumatologist (PRO)-based telemedicine follow (PRO-TR), nurse PRO-based telemedicine follow up (PRO-TN), or conventional physician follow up (control) for 1 year. The PRO-telehealth interventions achieved similar disease control as compared to those in the control conventional follow-up group (7). Similarly, Taylor-Gjevre et al. randomized 85 rheumatoid arthritis (RA) patients into either in-person or video conference-based telehealth follow up over a 9-month period and found out there were not significant differences in disease activity measurements and quality of life (8). These data support the equivalence of telerheumatology to in-person visits in achieving quality of care.

The current study assessed the patient and provider experiences with virtual care and evaluated healthcare utilization and quality of life among patients receiving virtual care during and after the peak of COVID-19 pandemic to provide more information on the effectiveness of telemedicine in rheumatology practices.

## Methods

This study is a longitudinal cohort study that evaluated the satisfaction with and effectiveness of telemedicine in a New York city Rheumatology academic practice. We designed two parallel seven-item questionnaires to evaluate the patient and provider experiences with virtual care. The question items were described in **Tables 2**, **3**, respectively. The two questionnaires, despite evaluating different sides of virtual care, contain similar items.

Patients with rheumatic diseases who received virtual care in a video conference format from a single rheumatology clinic in New York city during April 2020 to September 2020 were invited to participate in the survey. The seven-item Patient Questionnaire was disseminated to patients shortly through telephone or web links after they completed the virtual visit to evaluate their satisfaction and experience with the encounter; the socio-demographic data were collected through medical record review. The survey was repeated October 2021 to January 2022 with additional and optional EQ-5D-3L questionnaire to evaluate the quality of life. The EQ-5D-3L descriptive system comprises the following five dimensions: mobility, self-care, usual activities, pain/discomfort, and anxiety/depression. Each dimension has 3 levels: no problems, some problems, and extreme problems. The patient is asked to indicate his/her health state by ticking the box next to the most appropriate statement in each of the five dimensions. This decision results into a 1-digit number that expresses the level selected for that dimension. The digits for the five dimensions can be combined into a 5-digit number that describes the patient's health state. The scoring algorithm for the EQ-5D index is based on US community preferences (9). Data on the total number of in-person and telemedicine office visits, admissions were collected from electronic medical records.

Concurrently we surveyed 17 rheumatology healthcare providers from Columbia University Irving Medical Center who delivered virtual care in 2020 and in 2021 with the seven-item Provider Questionnaire.

The study protocol was approved by the Columbia University Irving Medical Center (CUIMC) Institutional Review Board (IRB).

## Results

### Socio-Demographic Information

100 out of 110 (91%) consecutive patients were able to complete the survey in 2020, and 100 out of 108 (92%) consecutive patients responded to the repeat survey in 2021. Additionally, 71 (66%) patients completed the EQ-5D-3L.

The socio-demographics of the 100 (90.9% of 110 and 92.6% out of 108) patients that responded to the survey in 2020 and 2021 are summarized in Table 1. Sixty-seven patients responded to both surveys in 2020 and 2021.

**Table 1:** Patient characteristics of the survey respondents.

**Characteristics**	**Categories**	**Year 2020, *n* (%)**	**Year 2021, *n* (%)**
Gender	Male	9 (9%)	7 (7%)
	Female	91 (91%)	93 (93%)
Age (years)	20–30	18 (18%)	16 (16%)
	31–40	22 (22%)	24 (24%)
	41–50	22 (22%)	23 (23%)
	51–60	21 (21%)	24 (24%)
	61–70	13 (13%)	10 (10%)
	71–80	4 (4%)	3 (3%)
Race	White	41 (41%)	50 (50%)
	Black or African American	25 (25%)	22 (22%)
	Asian	7 (7%)	11 (11%)
	Hispanic	26 (26%)	26 (26%)
Health insurance	Insured	100 (100%)	100 (100%)
	Uninsured	0 (0%)	0 (0%)
Employment Status	Employed	61 (61%)	54 (54%)
	Full-time students	7 (7%)	8 (8%)
	Unemployed	32 (32%)	38 (38%)
Diagnoses	Systemic lupus erythematosus (SLE)	60 (60%)	70 (70%)
	SLE with history of lupus nephritis	14 (23%)	17 (24%)
	Rheumatoid arthritis (RA)	7 (7%)	4 (4%)
	Undifferentiated connective tissue diseases (UCTD)	7 (7%)	5 (5%)
	Psoriatic arthritis (PsA)	5 (5%)	7 (7%)
	Sjogren's syndrome (SS)	4 (4%)	3 (3%)
	Spondylitis	3 (3%)	1 (1%)
	Other (sarcoidosis, myositis, osteoarthritis, fibromyalgia, uveitis, vasculitis)	14 (14%)	10 (10%)
EQ-5D-3L	Mobility *n* (%)	Level 1	51 (72%)
		Level 2	19 (27%)
		Level 3	1 (1%)
	Self-Care *n* (%)	Level 1	59 (83%)
		Level 2	12 (17%)
		Level 3	0 (0%)
	Usual Activities *n* (%)	Level 1	42 (59%)
		Level 2	26 (37%)
		Level 3	3 (4%)
	Pain/Discomfort *n* (%)	Level 1	17 (24%)
		Level 2	45 (63%)
		Level 3	9 (13%)
	Anxiety/Depression *n* (%)	Level 1	38 (54%)
		Level 2	28 (39%)
		Level 3	5 (7%)
	Index Score (Mean ± SD)	0.74 ± 0.20

Of the 100 patients surveyed in 2020, 91 (91%) were women with a mean ± standard deviation (SD) age of 44.3 ± 12.9 years; 41 (41%) were White, 25 (25%) African American, 26 (26%) Hispanic, and 7% Asian; 60 (60%) of the patients had a diagnosis of Systemic Lupus Erythematosus (SLE), and the remaining 40 (40%) had other systemic autoimmune diseases [undifferentiated connective tissue disorder (UCTD), rheumatoid arthritis (RA), psoriatic arthritis (PsA), and spondyloarthritis (SpA)].

Of the 100 patients surveyed in 2021, 93 (93%) were women with a mean ± SD age of 44.8 ± 13.0 years; 50 (50%) were White, 22 (22%) African American, 26 (26%) Hispanic, and 11% Asian; 70 (70%) of the patients had a diagnosis of Systemic Lupus Erythematosus (SLE), and the remaining 30 (30%) had other systemic autoimmune diseases [undifferentiated connective tissue disorder (UCTD), rheumatoid arthritis (RA), psoriatic arthritis (PsA), and spondyloarthritis (SpA)].

Interestingly, in these two cohorts of patients with rheumatic diseases and a large proportion of SLE patients, over 60% patients were employed or full-time students (Table 1).

Seventeen providers responded to the survey both in 2020 and 2021. The providers included 15 physicians and two nurse practitioners from Columbia University Irving Medical Center Division of Rheumatology; of the 15 physicians, nine were board-certified rheumatologists and six were rheumatology fellows in training.

### Survey Data

The survey results for patients and providers are shown in Tables 2, 3.

**Table 2:** Telemedicine questionnaire—patients.

**Questionnaire item (question type)**	**Response**	**Year 2020 *n* (%)**	**Year 2021 *n* (%)**
How satisfied were you with your previous telemedicine visit? (multiple-choice question, single answer)	Highly satisfied Satisfied Neither satisfied nor unsatisfied Not satisfied Highly unsatisfied	50 (50%) 11 (11%) 5 (5%) 0 (0%)	58 (58%) 36 (36%) 5 (5%) 1 (1%) 0 (0%)
Reasons for satisfaction?	Avoid coming into the office	73 (73%)	73 (73%)
(multiple-choice question,	Call went smoothly	77 (77%)	50 (50%)
multiple answers)	Decrease their concerns over condition, medications and risk of COVID-19	75 (75%)	45 (45%)
Reasons for unsatisfaction?	Technical difficulties	4 (4%)	2 (2%)
(multiple-choice question,	Visit was too short	2 (2%)	0 (0%)
multiple answers)	Visit was too basic for their needs	4 (4%)	3 (3%)
How comfortable were	Very comfortable	62 (62%)	76 (76%)
you with your previous telemedicine	Comfortable	24 (24%)	20 (20%)
visits? (multiple-choice question,	Neither comfortable nor uncomfortable	11 (11%)	2 (2%)
single answer)	Uncomfortable	3 (3%)	2 (2%)
	Highly uncomfortable	0 (0%)	0 (0%)
The physician was able to	Strongly agree	54 (54%)	62 (62%)
address what was bothering me	Agree	37 (37%)	31 (31%)
through the telemedicine visit? (multiple-choice question,	Don't know	5 (5%)	2 (2%)
single answer)	Disagree	4 (4%)	0 (0%)
	Strongly disagree	0 (0%)	0 (0%)
Overall, compared to an	Much better	10 (10%)	11 (11%)
in-person visit, the telemedicine	Better	6 (6%)	11 (11%)
visit was? (multiple-choice	Same	57 (57%)	60 (60%)
question, single answer)	Worse	25 (25%)	17 (17%)
	Much worse	2 (2%)	1 (1%)
I would have a telemedicine	Yes	77 (77%)	83 (83%)
appointment in the future, if	Unsure	14 (14%)	12 (12%)
given the option. (multiple-choice	No	9 (9%)	3 (3%)
question, single answer)		

**Table 3:** Telemedicine questionnaire—providers.

**Questionnaire item**	**Response**	***n* (%)**	***n* (%)**
How satisfied were you with your previous telemedicine visit?	Highly satisfied	0 (0%)	1 (5.9%)
(multiple-choice question, single answer)	Satisfied	3 (17.6%)	12 (70.6%)
	Neither satisfied nor unsatisfied	14 (82.4%)	1 (5.88%)
	Not satisfied	0 (0%)	3 (17.65%)
	Highly unsatisfied	0 (0%)	0 (0%)
Reasons for satisfaction? (multiple-choice question, multiple answers)	Able to work remotely	3 (17.6%)	0 (0%)
	Call went smoothly	3 (17.6%)	0 (0%)
	It helps patients access care	7 (41.2%)	7 (41.2%)
	It helps patients decrease anxiety	11 (64.7%)	5 (29.4%)
Reasons for unsatisfaction? (multiple-choice question,	Technical problems	12 (70.6%)	0 (0%)
multiple answers)	Difficult communication	15 (88.2%)	0 (0%)
	Lack of physical examination	14 (82.4%)	3 (17.6%)
	Complex coordination of care	8 (47.1%)	0 (0%)
How comfortable were you with your previous telemedicine visits?	Very comfortable	2 (11.8%)	7 (41.2%)
(multiple-choice question, single answer)	Comfortable	10 (58.8%)	5 (29.4%)
	Neither comfortable nor uncomfortable	5 (29.4%)	3 (17.7%)
	Uncomfortable	0 (0%)	1 (5.9%)
	Highly uncomfortable	0 (0%)	1 (5.9%)
I was able to address what was bothering the patients through the	Always	0 (0%)	1 (5.9%)
telemedicine visit. (multiple-choice question, single answer)	Sometimes	17 (100%)	16 (94.1%)
	Never	0 (0%)	0 (0%)
Overall, compared to an in-person visit, the telemedicine visit was?	Much better	0 (0%)	0 (0%)
(multiple-choice question, single answer)	Better	2 (11.8%)	1 (5.9%)
	Same	1 (5.9%)	2 (11.8%)
	Worse	14 (82.4%)	10 (58.8%)
	Much worse	0 (0%)	4 (23.5%)
Would you recommend telemedicine visit to other physicians?	Yes	12 (70.6%)	9 (52.9%)
(multiple-choice question, single answer)	Unsure	4 (23.5%)	6 (35.3%)
	No	1 (5.9%)	2 (11.8%)

Compared to 2020, patient respondents in 2021 reported a higher level of satisfaction (94 vs. 84%, *p* < 0.05). As expected, respondents felt more comfortable in 2021 with the telemedicine format (96 vs. 86%, *p* < 0.05). The majority of respondents (91% in 2020 and 93% in 2021) acknowledged that physicians were able to satisfactorily address the issues and concerns that prompted the visit. The percentage of respondents who considered the experience to be the same as the in-person experience remained high (57% in 2020 and 60% in 2021), more respondents in 2021 reported that telemedicine was better than in-person visit (22 vs. 16%). Finally, when asked whether they would use telemedicine in the future, 77% of 2020 respondents and 83% of 2021 respondents responded “yes.” In the 2021 survey we also asked if telemedicine was an acceptable or preferred alternative to the in-person visits; it was acceptable to 94 (94%) and preferred by 45 patients (45%).

Among the 17 providers surveyed in 2020, 3 (17.6%) expressed satisfaction with telemedicine while 14 (82.4%) felt that the telemedicine visits were inferior to conventional in-person clinic visits. In contrast, 13 (76.5%) of 17 providers in 2021 reported satisfaction with telemedicine but 14 (82.3%) still thought the telemedicine visits were worse or much worse than in-person visits. Noticeably, technical difficulties, unsatisfactory communications, and insufficient physical examinations were reported by 12 (71%), 15 (88%), and 14 (82%) providers in 2020 as reasons for dissatisfactions with virtual care. The lack of physical examination was the most reported reason for dissatisfaction with telemedicine among providers in 2021. However, 12 (70.6%) in 2020 and 9 (52.9%) in 2021 still chose to recommend telemedicine visits to other physicians.

### Quality of Care

The average number of in-person office visits, telemedicine visits, and admissions per survey respondent for 2019, 2020, and 2021 were shown in Table 4. There was no significant difference among the total number of visits per patient in 2019 (3.82 ± 2.85), 2020 (4.07 ± 2.59), and 2021 (4.38 ± 3.32). During 2020, the peak of COVID-19 pandemic, telemedicine visits constituted over 1/3 of total visits per year (1.49 ± 1.52), and their percentage (38.64% ± 33.66%) remained stable beyond the peak of pandemic into 2021 (35.64% ± 32.39%). There was no increase in the number of admissions, during and after the peak of pandemic (0.36 ± 0.83 in 2019, 0.4 ± 0.82 in 2020, and 0.48 ± 1.09 in 2021) despite the increased burden on the healthcare system in 2020 and 2021.

**Table 4:** Admission rate and volume of in-person and telehealth visits.

	**Year 2019**	**Year 2020**	**Year 2021**	***P-*** **value**	
				**2020 vs. 2019**	**2021 vs. 2020**
Total number of visits (mean ± SD)	3.82 ± 2.85	4.07 ± 2.59	4.38 ± 3.32	0.45	0.30
Total number of in-person visits (mean ± SD)	3.82 ± 2.85	2.58 ± 2.08	3.01± 3.19	<0.0001	0.05
Total number of telehealth visits (mean ± SD)	0	1.49 ± 1.52	1.37 ± 1.38	N/A	0.57
Percentage of telehealth visits (mean ± SD)	0	38.64% ± 33.66%	35.64% ± 32.39%	N/A	0.41
Admission per year (mean ± SD)	0.36 ± 0.83	0.4 ± 0.82	0.48 ± 1.09	0.61	0.51

### Quality of Life

The data on quality of life was collected only in 2021 using the EQ-5D-3L questionnaire. The numbers and percentages of patients (*n* = 71) reporting each level of problem on each dimension of the EQ-5D-3L are shown as Table 1. Our cohort has a mean EQ-5D-3L index score (± SD) of 0.74 ± 0.20, which is significantly lower than the US population norm (0.87) but similar to the pre-pandemic level reported among patients with SLE and connective tissue disorders (0.74) in the nationally representative Medical Expenditure Panel Survey (MEPS) (10). Additionally, 43 (60.6%) patients acknowledged that “telemedicine helped me when I was feeling down during the pandemic last year” and 46 (64.8%) agreed that “telemedicine helped to decrease my everyday stress over the past year.” Interestingly, only 5 of these 71 (7.0%) patients were “neither satisfied nor dissatisfied” or “not satisfied” with telemedicine and their mean EQ-5D index score was 0.70 ± 0.15 slightly lower than those who felt satisfied or highly satisfied with telemedicine (0.75 ± 0.20); additionally, 3 of 5 (60%) were unsure that telemedicine helped to address their anxiety or depression.

## Discussion

Telemedicine has now been extensively used in the management of rheumatology patients but literature examining its outcomes has been scarce. Before the COVID-19 pandemic, a few randomized clinical trials investigating the role of telemedicine were conducted in RA patients, which consistently reported high satisfaction rates of 80–90% (7, 8). More recently, six studies conducted among patients with rheumatic diseases during the COVID-19 pandemic indicated high acceptance and satisfaction with telemedicine for the delivery of rheumatology care (5). Multiple studies investigated factors associated with the acceptance of telemedicine by patients with rheumatic diseases. Ferucci et al. surveyed 56 patients seen by telemedicine (TM group) vs. 66 patients seen in-person (in-person only group), and reported factors associated with the use of telemedicine included a higher disease activity, a higher number of rheumatologist visits in the preceding year, a more positive perception of telehealth, and a visit with a physician who used telehealth more often (11). Breslau et al. found in a survey study of 2080 adults that participants were generally willing to use video visits but preferred in-person care (12). More recently, Moskowitz et al. proposed that patients have different expectations of providers in telemedicine based on their locus of control; and delivering tailored communication in telemedicine could enhance satisfaction (13).

Our cohort, with a high number of SLE patients, showed similar satisfaction rates (84% in 2020 and 94% in 2021) to those reported in the literature. However, the high level of frustration with telemedicine visits among healthcare providers in our center raises concerns as to whether disease activity can be assessed adequately using this format. In 2017, McDougall et al. published a systematic literature review which examined the use of telemedicine (video conferences and telephone consultations) in the diagnosis and/or management of inflammatory/autoimmune rheumatic diseases that included one randomized controlled trial and 19 observational studies. They concluded that there was limited evidence to support the effectiveness of telemedicine (14). Additionally, Han et al. reviewed data from three randomized clinical trials and three observational studies evaluating the role of virtual care in the management of patients with RA and reported equivalent control of disease activity and good patient experiences compared to conventional follow-up strategies (15).

Alexander et al. conducted a cross-sectional analysis of the US National Disease and Therapeutic Index Audit of 125.8 million primary care visits between January 2018 and June 2020. The authors reported that the pandemic was associated with a 25% decrease in primary care in-person office visits, which was in part due to the increase in telemedicine that accounted for 35.3% of encounters during the second quarter of 2020 (16). A similar percentage of telemedicine visits (38.6%) were observed in our cohort without a significant change in the total number of encounters. George et al. examined trends in in-person vs. telehealth visits among 300 rheumatology providers from 92 offices during COVID-19 and post-COVID-19 transition (May–August 2020); the authors found that telehealth increased substantially during the peak of the pandemic-−41.4% of all follow up visits (March to May 2020), and slightly decreased during the post-COVID-19 transition (27.7% of visits). In our cohort the percentage of telehealth visits remained stable in 2021 at 35.6% (17).

In a 2017 prospective study of hospitalizations among 155 Danish SLE patients, Busch et al. reported a crude hospitalization rate of 25% of the cohort per year and an annual admissions rate of 0.50 (18). Lawson et al. brought up a model of assessing outcomes of interest in SLE, which include healthcare utilization such as hospitalizations, disease activity, disease damage, mortality, and quality of life (19). Given the susceptibility of SARS-CoV-2 infection among patients with rheumatic diseases, the rate of hospitalization had the potential to be higher during the pandemic (20). Our cohort with a large proportion of SLE patients showed a stable hospitalizations rate (0.36, 0.40, 0.48/year in 2019, 2020, and 2021). Given the fact that 91% and 93% patients agreed or strongly agreed that “physicians were able to address what was bothering me during the telehealth visits,” we propose that the stable hospitalization rate during the COVID-19 pandemic may reflect the effectiveness of telehealth visits in addressing the medical needs of our patients; however, this assumption needs further investigation. Patients with rheumatic diseases in our cohort maintained a relatively stable outpatient healthcare utilization during the COVID-19 pandemic despite public health policies with non-significant increases in the total number of visits (3.82, 4.07, and 4.38 in year 2019, 2020, and 2021), which was almost all contributed by telerheumatology. However, it should be noted that the actual healthcare utilization rates might be underestimated given that both patients and physicians might attempt to maximally reduce the time spent in the hospital during the COVID-19 pandemic (21).

Patients with rheumatic diseases were reported to have significant impairment of health-related quality of life (HR-QoL) during the COVID-19 pandemic, especially in psycho-emotional dimension due to health care interruptions (22). However, our cohort showed quality of life similar to that reported pre-pandemic, as indicated by a stable EQ-5D-3L index score; and over 60% of survey respondents acknowledged that telemedicine helped to stabilize and improve their psycho-emotional status. Our clinic responded rapidly during the peaks of COVID-19 pandemic in 2020 and 2021; 96 % of total visits were switched to telemedicine as early as the last 2 weeks of March 2020. In fact, research personnel and medical students all helped keep patients connected with our clinic and optimized the transition to telemedicine. This swift transition to telemedicine in our rheumatology practice minimized the health care interruption experienced by other medical systems. While these data did not provide direct comparisons, we submit that telemedicine might have helped to improve patients' quality of life by increasing their access to rheumatology care and addressing their anxiety and/or depression about healthcare interruption.

As rheumatology clinical practices change, it is becoming clear that telemedicine will complement traditional face-to-face clinical encounters in care delivery and clinical trials. Disease activity indices such as the British Isles Lupus Assessment Group Index (BILAG) and the Systemic Lupus Erythematosus Disease Activity Index-2000 (SLEDAI-2K) for SLE, and the RA Disease Activity Score 28 (DAS-28) for RA are critical in clinical studies, and require physician assessments (23–25). Virtual disease activity indices have not yet been compared to face-to-face measures. Moreover, the physical examinations, especially musculoskeletal and cutaneous examinations, as essential components of patient evaluations, are difficult to conduct virtually (26, 27). The ability of current technology to allow for accurate evaluations by virtual visits is not yet fully understood. Accordingly, we and others have embarked on the task to further evaluate the role of telemedicine in disease activity assessments as virtual outcome measures might provide meaningful targets for optimizing treatment and assessing response in clinical trials.

We acknowledge several weaknesses of the study. First of all, the questionnaires used in the current study were not previously validated. Second, 67 of the 100 survey respondents in 2020 completed the survey again in 2021, the two cohorts in 2020 and 2021 were not the same, which limited our ability to perform a pairwise comparison. Third, EQ-5D-3L questionnaire was only disseminated to patients in 2021; as such, we were unable to directly compare 2020 and 2021 quality of life data of our survey respondents, which might bring in more persuasive data on the effectiveness of telerheumatology.

The data presented here support a high level of patient satisfaction with telerheumatology and suggest effectiveness in disease control and quality of care. More in depth evaluations of disease control, quality of care, and quality of life are needed to fully define the role of telemedicine in everyday rheumatology care.

## Data Availability Statement

The raw data supporting the conclusions of this article will be made available by the authors, without undue reservation.

## Ethics Statement

The studies involving human participants were reviewed and approved by Columbia University Irving Medical Center Institutional Review Board. Written informed consent for participation was not required for this study in accordance with the national legislation and the institutional requirements.

## Author Contributions

WT and SI wrote the first draft of the manuscript. AA, CA, MM, and MD contributed to the conception and design of the study. SI, JW, JB, and LK collected the data. WT, SI, JB, and LK performed the statistical analysis. All authors contributed to the manuscript revision, read, and approved the submitted version.

## Funding

This research was partially supported by the United States Department of Defense (DoD).

## Conflict of Interest

The authors declare that the research was conducted in the absence of any commercial or financial relationships that could be construed as a potential conflict of interest.

## Publisher's Note

All claims expressed in this article are solely those of the authors and do not necessarily represent those of their affiliated organizations, or those of the publisher, the editors and the reviewers. Any product that may be evaluated in this article, or claim that may be made by its manufacturer, is not guaranteed or endorsed by the publisher.
